# Integrating traditional medicine into the African healthcare system post-Traditional Medicine Global Summit: challenges and recommendations

**DOI:** 10.11604/pamj.2024.47.146.43011

**Published:** 2024-03-27

**Authors:** Martin Ikhoyameh, Wealth Egbeaduma Okete, Ruth Mosunmola Ogboye, Olaitan Kayode Owoyemi, Ololade Samson Gbadebo

**Affiliations:** 1 Department of Biochemistry, University of Benin, Benin City, Nigeria,; 2 Department of Pharmaceutical Chemistry, Obafemi Awolowo University, Ile-Ife, Nigeria,; 3 Department of Biological Sciences, Ajayi Crowther University, Oyo, Nigeria

**Keywords:** Traditional medicine, Africa, World Health Organization, healthcare

## Abstract

The World Health Organization (WHO) held the inaugural Traditional Medicine Global Summit in India in 2023 to promote the evidence-based integration of traditional medicine (TM) into the global healthcare system. This summit offers many opportunities for Africa, where most people rely on TM for health care. TM is widely accepted and affordable in the region but faces many challenges that limit its potential. This article identifies some of the most pressing challenges to incorporating TM into standard healthcare in Africa. It also proffers useful recommendations on how these issues may be addressed while identifying key stakeholders whose contributions may hamper or enhance the realization of WHO´s goals for TM in the continent.

## Commentary

The first Traditional Medicine Global Summit was held in Gujarat, India, in August 2023, under the auspices of the World Health Organization (WHO). The summit aimed to promote the evidence-based integration of Traditional Medicine (TM) into the global healthcare system, as part of the WHO´s new vision for this field [1]. This was not an isolated event, but rather a culmination of a series of initiatives and efforts by various countries, including African countries, over the past century. Following the Alma-Ata Declaration of 1978, which advocated for primary healthcare as a human right, the Declaration of Astana 2018 included the appropriate use of TM as one of the strategies to achieve universal health coverage. The WHO conducted three surveys between 2005 and 2018 to monitor the trends and developments in the use of traditional and complementary medicine among its member states. The surveys revealed an increasing number of countries that had adopted national policies and regulations on TM [2]. Furthermore, in 2003, African leaders declared an African Traditional Medicine (ATM) day to raise awareness and recognition of the role of TM in improving health outcomes. The ATM Day was endorsed by the WHO African Region in 2015 as a key component of its regional strategy on TM [3].

Traditional medicine refers to the “knowledge, skills, and practices that are based on the theories, beliefs, and experiences of different cultures, whether explicable or not, and that are used to maintain or improve health, or to prevent, diagnose, or treat physical and mental illnesses” [4]. TM has a long history and wide acceptance in Africa, especially among rural communities who find it more affordable. ATM encompasses indigenous practices and therapies, from plants and animal parts, used to treat, diagnose, or manage diseases in the continent. Herbal therapies, which represent the most common form of TM in the continent, are used by up to 80% of the African populace [5]. Gaps created by limited access to essential medicines, poor health infrastructure, and dissatisfaction with orthodox medicine have promoted the use of these therapies, particularly in rural communities. Their affordability alongside individual religious affiliations or cultural beliefs fills these gaps sufficiently and contributes to the wide use of herbal remedies across the continent. However, TM in Africa faces some challenges and criticisms from the elites who question its standardization, quality, and safety. Although the practice varies according to ethnic groups, there are some commonalities in the use of plant and animal-based remedies in African countries, such as Ghana, Zambia, Tanzania, South Africa, Kenya, and Nigeria. Despite efforts to integrate TM into the healthcare system of countries like Ghana, Mali, Benin, and Nigeria [3], the impact of these initiatives is still limited.

Traditional Medicine (TM) has achieved remarkable success in some Asian countries, like China and India, where it is well-integrated with conventional medicine in various levels of health institutions. The scientific evidence supporting the efficacy and safety of TM for health improvement is growing rapidly. Moreover, about 40% of the pharmaceuticals in the market are derived from natural sources [1]. Considering this evidence, the WHO´s recent initiative to mobilize global political will and support for TM is a welcome development. However, there are some regional and demographic challenges that may hinder the implementation of this initiative.

### These challenges encompass

**Lack of necessary resources and scientific validation:** despite the increasing national recognition of TM in many African regions, its incorporation into systematic healthcare practice is hindered by several factors. The major overarching barriers include the lack of resources and effective policies to facilitate the integration. While a significant proportion of ATM is deeply rooted in cultural and spiritual practices, there is a lack of adequate scientific validation. Additionally, the necessary resources for research in addressing this challenge are lacking.

**Scarcity of trained traditional health workers:** the paucity of well-trained health workers to advocate and pioneer the integration of ATM into systematic health care is one offshoot of the aforementioned challenges. ATM is primarily informal and said to not require formal education or professional training to practice. Consequently, it is vulnerable to fraud and quackery, and practitioners′ unproven medical claims are encouraged to thrive. These features of the practice incite a lack of trust in TM among the general public and conventional medicine practitioners.

**Absence of training in traditional medicine:** according to a review by Innocent, merely a handful of formal institutions in sub-Sahara Africa offer training in TM [6]. This could also be attributed to the government´s questionable commitment to regularly develop TM. For instance, in 2020, the Nigerian government inaugurated a council to focus on establishing institutions and programs to enable research and explore values in TM. However, there have been no reported outcomes from this initiative. The unavailability of thriving TM educational programs for both modern medicine providers and the general public has posed a barrier to healthy coexistence and mutual understanding with TM practitioners.

**Incorporation of spirituality:** the integration of spiritual elements such as incantations or sacrifice with many ATM is a strong barrier to collaborations between orthodox and traditional systems. While the blend of spirituality and medicine is acceptable to some indigenous users, it constitutes a challenge to individuals of other religious dispositions, since they may consider such remedies ineffective or simply diabolic.

**Knowledge transfer:** effective knowledge sharing and the ethical use of intellectual property are crucial to the growth of organized science. However, most ATM practitioners neither practice proper documentation nor observe a methodical approach to knowledge sharing. Instead, knowledge is commonly treated in an esoteric manner and shared orally to subjectively selected individuals in ATM. This transfer of knowledge is done casually often spanning years and potentially contributing to knowledge loss given the possibility of death. Furthermore, the fear of creating competition sometimes surpasses the desire to provide care leading to knowledge hoarding. The unethical use of knowledge obtained from traditional practitioners by scientists without proper acknowledgment presents an extra reason for the increased reluctance of some traditional practitioners to share their knowledge.

**Cultivation concerns:** in addition to the knowledge of TM not being conserved over generations, the diversity of medicinal plants over time is also threatened by human factors. There are rising concerns about the effect of harvesting rate on the scarcity of some plant species. While not all plant species are endangered, some are susceptible to rarity due to overexploitation, uncontrolled collection, and deforestation [7]. Moreover, there is a lack of information on how various commercially valuable medicinal plants respond to cultivation methods. TM users often view cultivated medicinal plants with skepticism because they understand the impact of the environment on the “medicinal power” of plants.

For the successful integration of TM into the African health system, we put forward the following recommendations.

**Bonus system for referrals and knowledge sharing:** in Ghana, co-referral systems between traditional and orthodox medicine practitioners have contributed to the co-integration of the practices [8]. Following a similar model, the national umbrella of ATM practitioners should develop a user-friendly and easy referral system that rewards traditional healers with incentives for referring sick patients to the hospital. This will reduce the risk of death associated with the lack of proper diagnosis and treatment.

To address knowledge hoarding or loss, pharmaceutical industries in Africa could set up programs for training traditional healers on the importance of knowledge sharing. Traditional healers who are willing to share their knowledge are then rewarded with incentives and intellectual property filings to encourage others to follow suit. Moreover, patency laws should be made to spur and encourage such services. Scientists can explore the knowledge shared to discover and develop novel therapeutic agents from medicinal plants.

**Education and advocacy:** most rural dwellers in African countries are unaware of the risks associated with self-medicating with herbal medicine and consuming unregulated herbal concoctions. There is a need to properly communicate these life-endangering risks to uneducated locals. To adequately educate rural dwellers on the health hazards and risks associated with the consumption of herbal concoctions, the federal ministries of health should deploy teams of skilled science communicators. By developing and using context-specific and relatable approaches to passing scientific information tailored to each specific ethnic group, the science communicators will take responsibility for ensuring that the right information is communicated in a way that uneducated rural dwellers can relate to.

**Collaborations between traditional and modern medicine practitioners:** forming a joint association between traditional and modern medicine practitioners is a strategy that will produce positive results. Although research suggests that medical professionals tend to be less interested in engaging their traditional counterparts [9], it will be helpful to foster such cooperation. Collaboration will reduce conflicts and issues arising from the current practice of the two groups working in silos and stifling knowledge sharing despite their goal of improving healthcare. Accordingly, a common ground for collaboration will be created that allows each group to identify their strengths and weaknesses and explore opportunities for improvement. By establishing regulatory policies, rules, and codes of conduct, such a joint association will further foster cooperation and address unethical practices. These laws could also detail the procedure for how herbal products may be used as a basis for managing diseases in orthodox settings, synthesizing new drugs, or managing medication side effects. Furthermore, collaboration may also help scientists to unravel, where necessary, or exclude the spiritual components of TM.

**Disciplinary measures to tackle quackery:** the government should sign or enforce existing legislation and implement a legal framework to penalize quacks and offenders who fail to refrain from administering herbal concoctions that have not been approved by the national agencies responsible for regulating and controlling the production and distribution of herbal medicine. The development and implementation of such policies can also fall under the purview of the joint association previously mentioned.

**Establishment of training programs in traditional medicine:** training programs, particularly those focused on educating and certifying traditional healers, should also be adopted to improve the practice of TM in Africa. Such programs must integrate the perspectives and expertise of both traditional and orthodox specialists to strengthen collaboration, promote knowledge sharing, and boost overall confidence in traditional medicine. This way, they may lay the groundwork for the documentation of existing and novel herbs with therapeutic potential, discovery, or standardization of new drug regimens as well as design of trials to test the efficacy or safety of herbal medications.

**Conservation, cultivation, and proper handling of herbs:** the conservation of medicinal plant diversity is important to achieve an efficient integration of herbal medicine into the African healthcare system. Adoption of conservation strategies - including working natural reserves, wild nurseries, botanical gardens seed banks, and the advocacy for good agricultural practices (GAP) - will help to improve species richness of medicinal plants in Africa [7]. As soil type and growth site of herbs have been shown to impact their phytochemical composition [10], advances in plant biotechnology may offer various avenues for cultivating plants without losing some of these useful constituents. Scientists should work with traditional healers to spot and study the natural habitats of specific herbs with medicinal value. Knowledge gained from such exploration may in turn be channelled into improving the yield of such plants in their natural habitats or domesticating them by designing mimic habitats that can match the natural ones with considerable similarity. While genetic manipulation may hold some value, it is important to consider its perceived benefits and drawbacks and involve relevant stakeholders before applying it to TM.

In conclusion, TM enjoys a relatively wide patronage across Africa. However, its potential to improve the health status of the continent´s populace remains largely underexplored. Several challenges, which implicate both traditional and modern medicine practitioners as well as governments, contribute to this problem. We have proposed bonus systems, education and advocacy, collaboration, training programs, and disciplinary measures as approaches to addressing these challenges. Overall, we argue that the longstanding prejudice against TM by conventional medical practitioners will not change without policies that encourage research and advancement of TM in the continent.

## References

[1] Burki T (2023). WHO´s new vision for traditional medicine. Lancet.

[2] World Health Organization (2019). WHO Global Report on Traditional and Complementary Medicine 2019.

[3] Emeje M, Oppong Bekoe E, Graz B, Willcox M (2023). Traditional Medicine Development in Africa: Opinion. J Integr Complement Med.

[4] World Health Organization (2013). WHO Traditional Medicine Strategy 2014-2023.

[5] World Health Organization (2022). African Traditional Medicine Day 2022: Message of WHO Regional Director for Africa, Dr Matshidiso Moeti.

[6] Innocent E (2016). Trends and challenges toward integration of traditional medicine in formal health-care system: Historical perspectives and appraisal of education curricula in Sub-Sahara Africa. J Intercult Ethnopharmacol.

[7] Chen SL, Yu H, Luo HM, Wu Q, Li CF, Steinmetz A (2016). Conservation and sustainable use of medicinal plants: problems, progress, and prospects. Chin Med.

[8] Kwame A (2021). Integrating Traditional Medicine and Healing into the Ghanaian Mainstream Health System: Voices From Within. Qual Health Res.

[9] Lampiao F, Chisaka J, Clements C (2019). Communication Between Traditional Medical Practitioners and Western Medical Professionals. Front Sociol.

[10] Mangoale RM, Afolayan AJ (2020). Comparative Phytochemical Constituents and Antioxidant Activity of Wild and Cultivated *Alepidea amatymbica* Eckl & Zeyh. Biomed Res Int.

